# Isolation and Characterization of Werneria Chromene and Dihydroxyacidissimol from *Burkillanthus* *malaccensis* (Ridl.) Swingle

**DOI:** 10.3390/plants11111388

**Published:** 2022-05-24

**Authors:** Masyitah Zulkipli, Nuzum Mahbub, Ayesha Fatima, Stefanie Lim Wan-Lin, Teng-Jin Khoo, Tooba Mahboob, Mogana Rajagopal, Chandramathi Samudi, Gheetanjali Kathirvalu, Nor Hayati Abdullah, Ana Rita Pinho, Sonia M. R. Oliveira, Maria de Lourdes Pereira, Mohammed Rahmatullah, Anamul Hasan, Alok K. Paul, Mark S. Butler, Muhammad Nawaz, Polrat Wilairatana, Veeranoot Nissapatorn, Christophe Wiart

**Affiliations:** 1 School of Pharmacy, University of Nottingham Malaysia Campus, Semenyih 43500, Malaysia; khyy6mzz@nottingham.edu.my (M.Z.); hyxnm1@nottingham.edu.my (N.M.); hyysl1@nottingham.edu.my (S.L.W.-L.); tengjin.khoo@nottingham.edu.my (T.-J.K.); 2 Beykoz Institute of Life Sciences and Biotechnology, Bezmialem Vakif University, Istanbul 34093, Turkey; afatima@bezmialem.edu.tr; 3 Department of Medical Microbiology, University of Malaya, Kuala Lumpur 50603, Malaysia; tooba666@hotmail.com (T.M.); chandramathi@um.edu.my (C.S.); geetha.medmicrob@gmail.com (G.K.); 4 Faculty of Pharmaceutical Sciences, UCSI University, Kuala Lumpur 56000, Malaysia; mogana@ucsiuniversity.edu.my; 5 Natural Product Division, Forest Research Institute Malaysia (FRIM), Kepong 52109, Malaysia; norhayatiab@frim.gov.my; 6 Department of Medical Sciences, University of Aveiro, 3810-193 Aveiro, Portugal; arapinho@ua.pt (A.R.P.); mlourdespereira@ua.pt (M.d.L.P.); 7 Neuroscience and Signaling Laboratory, Institute of Biomedicine-IBIMED, University of Aveiro, 3810-193 Aveiro, Portugal; 8 CICECO-Aveiro Institute of Materials, University of Aveiro, 3810-193 Aveiro, Portugal; sonia.oliveira@ua.pt; 9 Hunter Medical Research Institute (HMRI), New Lambton Heights, NSW 2305, Australia; 10 Department of Biotechnology & Genetic Engineering, University of Development Alternative, Lalmatia, Dhaka 1207, Bangladesh; rahamatm@hotmail.com (M.R.); anamulhasanoris@gmail.com (A.H.); 11 School of Pharmacy and Pharmacology, University of Tasmania, Hobart, TAS 7001, Australia; alok.paul@utas.edu.au; 12 Institute for Molecular Bioscience, University of Queensland, Brisbane, QLD 4072, Australia; m.butler2@imb.uq.edu.au; 13 Department of Nano-Medicine, Institute for Research and Medical Consultations (IRM), Imam Abdulrahman Bin Faisal University, Dammam 34212, Saudi Arabia; nawwaz@gmail.com; 14 Department of Clinical Tropical Medicine, Faculty of Tropical Medicine, Mahidol University, Bangkok 10400, Thailand; 15 School of Allied Health Sciences, World Union for Herbal Drug Discovery (WUHeDD), Research Excellence Center for Innovation and Health Products (RECIHP), Walailak University, Nakhon Si Thammarat 80160, Thailand; 16 Institute for Tropical Biology and Conservation, Universiti Malaysia Sabah, Jalan UMS, Kota Kinabalu 88400, Malaysia

**Keywords:** *Burkillanthus malaccensis*, antibiotic potentiator, werneria chromene, dihydroxyacidissiminol, SARS-CoV-2, cathepsin L, nsp13 helicase, spike protein

## Abstract

The secondary metabolites of endemic plants from the Rutaceae family, such as *Burkillanthus* *malaccensis* (Ridl.) Swingle from the rainforest of Malaysia, has not been studied. *Burkillanthus* *malaccensis* (Ridl.) Swingle may produce antibacterial and antibiotic-potentiating secondary metabolites. Hexane, chloroform, and methanol extracts of leaves, bark, wood, pericarps, and endocarps were tested against bacteria by broth microdilution assay and their antibiotic-potentiating activities. Chromatographic separations of hexane extracts of seeds were conducted to investigate effective phytochemicals and their antibacterial activities. Molecular docking studies of werneria chromene and dihydroxyacidissiminol against SARS-CoV-2 virus infection were conducted using AutoDock Vina. The methanol extract of bark inhibited the growth of *Staphylococcus* *aureus*, *Escherichia* *coli*, and *Pseudomonas* *aeruginosa* with the minimum inhibitory concentration of 250, 500, and 250 µg/mL, respectively. The chloroform extract of endocarps potentiated the activity of imipenem against imipenem-resistant *Acinetobacter* *baumannii*. The hexane extract of seeds increased the sensitivity of *P. aeruginosa* against ciprofloxacin and levofloxacin. The hexane extract of seeds and chloroform extract of endocarps were chromatographed, yielding werneria chromene and dihydroxyacidissiminol. Werneria chromene was bacteriostatic for *P.* *aeruginosa* and *P.* *putida*, with MIC/MBC values of 1000 > 1000 µg/mL. Dihydroxyacidissiminol showed the predicted binding energies of −8.1, −7.6, −7.0, and −7.5 kcal/mol with cathepsin L, nsp13 helicase, SARS-CoV-2 main protease, and SARS-CoV-2 spike protein receptor-binding domain S-RBD. *Burkillanthus* *malaccensis* (Ridl.) Swingle can be a potential source of natural products with antibiotic-potentiating activity and that are anti-SARS-CoV-2.

## 1. Introduction

Nowadays, clinicians are confronted with the huge burden of treating patients infected by multidrug-resistant nosocomial bacteria [1,2]. Nosocomial bacteria, such as *Acinetobacter* *baumannii* and *Pseudomonas* *aeruginosa*, in intensive care units resist the main classes of antibiotics, such as oxazolidinooxazolidinones, lipopeptides, macrolides, fluoroquinolones, tetracyclines, β-lactams, β-lactam-β-lactamase inhibitor combinations, carbapenems, and glycopeptides antibiotics [3,4,5,6]. Besides bacterial species, drug-resistant parasites have emerged, such as *Plasmodium* species [7] and Brugia species [8].

Another challenge is the treatment of zoonotic viruses, such as the severe acute respiratory syndrome-associated coronavirus (SARS-CoV), the Middle-East respiratory syndrome-associated coronavirus (MERS-CoV), and the severe acute respiratory syndrome-associated coronavirus 2 (SARS-CoV-2) [1,2]. Since December 2019, SARS-CoV-2, the causative agent of the coronavirus disease 2019 (COVID-19) pandemic, has been infecting millions of people globally [9,10,11]. SARS-CoV-2 is an enveloped, monopartite, linear, single-stranded (+)-RNA zoonotic virus in the Coronaviridae that replicates in the lower respiratory tract of humans, leading in some cases to lethal pneumonia [3,12]. Vaccines have been developed, affording good protection rates, but there is still the need to develop leads to be taken orally for the prevention and/or treatment of COVID-19, as complementary to vaccination. Further, if the human pressure on the environment is not halted, other new zoonotic viruses are bound to emerge, for which an armamentarium of anti-viral molecules is required to attempt to limit casualties.

SARS-CoV-2 infects cells expressing the surface receptors angiotensin-converting enzyme 2 (ACE2) and cellular serine protease TMPRSS2 (transmembrane protease serine 2). SARS-CoV-2 enters the host cells by attaching its surface spike proteins to the surface ACE2 of human host cells [9,11,13]. TMPRSS2 and cysteine protease cathepsin L are also required to facilitate host cell entry. After fusion of the viral membrane with the host cell membrane, the viral genomic material is released in the cytoplasm and replicated, via several enzymes, including Nsp13 helicase [10,11,14,15,16]. Targeting these three proteins (ACE2, cathepsin L, Nsp13 helicase) can assist in the development of anti-COVID-19 drugs [11]. COVID-19 infection can progress to acute respiratory distress syndrome (ARDS) that favors secondary bacterial infection, leading to sepsis and, ultimately, death [11,17,18,19].

The alternative for the discovery of new antibiotics is to develop antibiotic-potentiators, also known as antibiotic adjuvants. Therefore, antibiotics, antibiotic-potentiators, and anti-viral compounds (more so against COVID-19 are urgently needed. Furthermore, since promising leads for new drugs have usually come from the plant kingdom [20,21], it becomes more important to study plants to help discover novel drugs that can play important therapeutic/prophylactic roles against newly emerging viruses and antibiotic-resistant microbial species. Unfortunately, various plants are becoming extinct for many reasons, including irresponsible deforestation, an extension of human habitat, conversion of forests and water bodies into agricultural land, and global warming [22].

The primary rainforests of Malaysia used to be one of the hotspots of plant biodiversity. A study carried out in 2008 found that a randomly chosen 5-hectare area in Ayer Hitam Forest, Puchong, contained 6621 trees belonging to 319 species in 148 genera and 51 families [23]. These plants can be a source of diverse secondary metabolites, which are yet to be explored [24]. As various therapeutic medications originate from the secondary metabolites (phytochemicals) of plants, any extinction or even rarity of plant species may make it impossible to ever know of the phytochemical properties of any given plant. Since these rainforests of Malaysia are steadily declining because of various commercial interests [25,26,27], the chances of discovering new drugs from plant species, any of which may be novel antibiotics, antibiotic-potentiators, or anti-viral drugs, are also decreasing rapidly. Thus, there is an urgent need to study these rainforest plants.

*Burkillanthusmalaccensis* (Ridl.) Swingle. (synonym: Citrus malaccensis Ridl.), in the family Rutaceae, is a low-land primary rainforest tree known to the Malays as “*limau hantu*” (meaning the lemon tree of ghosts) and commonly known as Burkill’s lime tree [28,29,30,31,32,33,34]. The current study investigated for the development of safe, effective, and inexpensive plant-based antibiotic potentiators that can improve current treatment strategies for treating bacteria-resistant infections and simultaneously can contribute to the development of anti-SARS-CoV-2 agents. The aims of this study were to examine the antibacterial properties of *B. malaccensis* leaves, bark, wood, and fruits (exocarp, endocarp, and seeds) against *Enterococcus* *faecium*, *Staphylococcus* *aureus*, *Klebsiella* *pneumoniae*, *Acinetobacter* *baumannii*, *Pseudomonas* *aeruginosa*, *Pseudomonas* *putida,* and *Enterobacter* spp.) [35]; to examine the antibiotic-potentiating properties of extracts with antibiotics, to isolate the major constituents in the active extracts, and to test their antibacterial effects in vitro and anti-SARS-CoV-2 effects in silico against (a) a complex of SARS-CoV-2 spike protein (S) receptor-binding domain (RBD) bound with human receptor angiotensin-converting enzyme 2 (hACE2) or S-RBD-hACE2; (b) cathepsin L; (c) SARS-CoV-2 Nsp13 helicase; (d) SARS-CoV-2 main protease Mpro (plays an integral part in viral replication); and (e) S-RBD. While ACE-2 is the receptor for the viral spike protein S, cathepsin L cleaves the spike protein and enhances the entry of virus [36], and SARS-CoV-2 Nsp13 possesses NTPase (nucleotide triphosphatase) and RNA helicase activities [37]. In this study, a plant extract that has not been investigated for its antiviral activities yielded two isolated compounds. Thus, it was of interest to study the antiviral activities against some SARS-CoV-2 proteins and their human targets so as not to miss out on anything of importance, but instead get a composite picture of the anti-SARS-CoV-2 potential of the isolated novel compounds.

## 2. Material and Methods

### 2.1. Plant Collection

Chemotaxonomic collection of *B*. *malaccensis* from Manong village close to the Kuala Kangsar Forest, State of Perak, Malaysia (4.7746° N, 100.9520° E), was performed in February 2017. It is a beautiful plant with thorny stems, trifoliate leaves, large white flowers, and large grapefruit-like fruits, containing numerous seeds covered with a yellow resin (personal observation). Samples of leaves, bark, wood, seeds, and fruits were collected. A voucher herbarium specimen with vernacular names, collection localities, and dates were deposited (Voucher number NB0541) for identification by taxonomists at the Forest Research Institute of Malaysia (FRIM). The use of plants in the present study complies with international guidelines. The plant was selected in fieldwork according to the following three criteria: (i) chemotaxonomic features; (ii) the presence of fruits or flowers to allow an accurate botanical identification; and (iii) a sufficient amount available.

### 2.2. Preparation of Plant Extracts

The collected leaves, bark, wood, seeds, endocarps (sac juice), and fruit pericarps from *B*. *malaccensis* were separated and air-dried at room temperature for two weeks. The dry materials were then finely pulverized by grinding using an aluminum collection blender (Philips, Guangdong, China); the powders obtained were weighed with a top-loading balance (Sartorius AG, Göttingen, Germany). Dried plant powders (200 g) were successively soaked at room temperature with hexane, chloroform, and methanol. Each extraction was performed using the maceration technique with a plant powder-to-solvent ratio of 1:5 (*w*/*v*) for 3 days at room temperature with 3 successive repetitions. The liquid extracts were subsequently filtered through qualitative filter papers, No. 1 (Whatman International Ltd., Maidstone, UK), using an aspirator pump (EW-35031-00, 18 L/min, 9.5 L Bath, 115 VAC), and the filtrates were concentrated to dryness under reduced pressure at 40 °C using a rotary evaporator (Buchi Labortechnik AG, Flawil, Switzerland). The dry extracts obtained were weighed and stored in tightly closed glass scintillation vials (Kimble, NY, USA) at −20 °C until further use.

The yields of extracts were calculated using the following formula:

% yield = ((Mass of dried extract)/(Mass of dried plant part)) × 100%

### 2.3. Tested Bacterial Strains

Stock cultures of bacteria used for this study were kindly provided by the Department of Medical Microbiology, Faculty of Medicine, University of Malaya, Malaysia. The following bacteria were used as test organisms from the American Type Culture Collection (ATCC, Manassas, VA, USA): *S. aureus* (ATCC11632), *B. subtilis* (ATCC 6633), *E. coli* (ATCC 25218), *P. aeruginosa* (ATCC 10145), and *A. baumannii* (clinical stain, imipenem-resistant) were sub-cultured in nutrient agar. *Pseudomonas putida* (ATCC 49128) was used for the antibacterial testing of isolated compounds. All sub-cultured bacterial specimens were aseptically transferred using an inoculating loop and prepared in 10 mL suspensions of Mueller-Hinton Broth (Oxoid, Hampshire, UK) and were used 15 min after inoculation. A fraction equivalent to 1 mL of the bacterial suspensions was transferred to a cuvette and subjected to a spectrophotometer (Biochrom, Cambridge, UK), where the UV absorbance value was monitored to be in the range of 0.008 to 0.10 at 625 nm, in order to be adjusted to 0.5 McFarland turbidity standards (Healthlink, Orlando, FL, USA), which correspond to a bacterial cell count of 1.5 × 10^8^ CFU/mL [38]. The extracts and phytoconstituents were prepared by dissolving in 10% DMSO and in a minimum essential medium to make a stock solution with a final concentration of 1% DMSO. This stock solution was diluted in liquid broth to further to obtain concentrations ranging from 500 to 5000 µg/mL.

### 2.4. Broth Microdilution Assay

Minimum inhibitory concentration (MIC) values were determined according to the guidelines of the Clinical and Laboratory Standards of the Institute. Briefly, bacterial strains were grown for 18–24 h at 37 °C. Colonies were directly suspended in cautiously adjusted Müller–Hinton broth (CAMHB) and adjusted to OD625 0.08–0.1, which corresponds to 1~2 × 10^8^ CFU/mL, followed by 10-fold serial dilutions to give 1 × 10^6^ CFU/mL. Bacterial suspensions (1000 µL) were added to the 96-well round-bottom microtiter plates. Each well was then filled with 100 µL of liquid broth containing extracts of phytoconstituents to yield final concentrations of extracts or phytoconstituents of 250, 625, 1000, 1500, and 2500 µg/mL, respectively. The 96-well plates were incubated for 24 h at 37 °C. The MIC was defined as the lowest concentration of extract or phytoconstituents that completely inhibited the growth of bacteria. Negative controls consisted of bacterial suspensions (100 µL) added to 96-well round-bottom microtiter plates containing 100 µL of liquid broth with a minimum amount of DMSO, as in extracts of phytoconstituent stock solution preparations. Minimum bactericidal concentration (MBC) was determined (for extracts with MIC values equal to or less than 250 µg/mL) by sub-culturing the test dilutions onto a sterile agar plate and further incubated for 18–24 h. The highest dilution that yielded 0% bacterial growth on agar plates was taken as the MBC, MIC and MBC values were calculated as the mean of triplicate experiments. Chloramphenicol, tetracycline, and imipenem were used as positive control antibiotics.

### 2.5. Antibiotic-Potentiating Assay

The ability of *B*. *malaccensis* extracts to increase the sensitivity of bacteria to antibiotics was measured by the technique described by Boonyanugomol et al., with slight modifications [39]. Standard antibiotic discs of ampicillin (10 µg/disc), gentamicin (10 µg/disc), imipenem (10 µg/disc), levofloxacin (5 µg/disc), penicillin G (10 µg/disc), and ciprofloxacin (5 µg/disc) (Sigma-Aldrich, St. Louis, MO, USA) were loaded with 10 µL of a 100 µg/µL solution of extracts of leaves, bark, wood, seeds, endocarps, or pericarps. The zones of inhibition were measured after overnight incubation (12 h) and estimated as follows:

1. Zone of combined extract and antibiotic > zone of extract + zone of antibiotic: synergy.
2. Zone of combined extract and antibiotic = zone of extract + zone of antibiotic: additive.
3. Zone of combined extract and antibiotic < zone of extract + zone of antibiotic: antagonism.

### 2.6. Cytotoxicity Assay

To study in vitro the cytotoxicity of extracts against human fibroblast cells (MRC-5 cell line, MRC-5 ATCC CCL-171 Homo sapiens lung normal), the reduction of (3-(4,5-dimethylthiazol-2-yl)-2,5-diphenyltetrazolium bromide) by a colorimetric assay (MTT) was used [40]. The extracts were prepared by dissolving in DMSO and in a minimum essential medium (MEM) to make a stock solution with a final concentration of 1% DMSO. This stock solution was diluted further to obtain concentrations ranging from 0 to 200 µg/mL. Cells were cultured in Rosewell Park Memorial Institute media (RPMI) supplemented with 10% Fetal Bovine Serum (FBS). Cells were incubated with the diluted plant extracts for 48 h at 37 °C in 5% CO_2_. After cells were washed twice with saline, a solution of MTT (0.5 mg/mL) in phosphate buffer saline (PBS) was added to the wells. After 4 h of incubation, the wells were washed, and the formazan residue dissolved in DMSO (0.1 mL per well). The absorbance was then measured in a spectrophotometer (SpectraMax M3, Multi-Mode Microplate reader, Molecular Devices, San Jose, CA, USA) at a wavelength of 570 nm and plotted against the concentration of the extracts. Cells with no added test reagents were taken as untreated cells with 100% viability, and cells with RPMI 1640 medium were used as blanks. The cells viability percentage was plotted against the extract’s concentration. All experiments were performed in triplicate, and results were expressed as the concentration by reducing the number of live MRC-5 cells by 50% (CC_50_). The percentage of viability was calculated using the following formula:

% Viability = [(Abs sample − Abs blank)/(Abs untreated − Abs blank)] × 100%

The effects of extracts on the % viability of cells (*y*-axis) was plotted against log concentrations (g/L) (*x*-axis) and interpolated sigmoidal curves (4-parameter logistic curve, 4 PL) using GraphPad Prism and the CC_50_ was automatically determined using GraphPad Prism v6 software (GraphPad Software Inc., La Jolla, CA, USA).

### 2.7. Isolation and Identification of Compounds

Hexane extract from seeds (8.5 g) was loaded onto preparative thin-layer chromatography (TLC) plates in a mobile phase of hexane: ethyl acetate: dichloromethane (40:30:30). TLC analysis revealed the presence of a major layer under UV light (254 nm) with a retention factor (Rf) value of 0.25. TLC layers were stained with vanillin sulfuric acid. The layer with the lowest purple Rf stained with vanillic sulfuric acid was collected, redissolved, filtered, and isolated to yield the compound 1 (5 mg) that was identified as werneria chromene (C_15_H_16_O_3_, molecular mass = 244.28 g/mol) by comparing its ^1^H-NMR (proton nuclear magnetic resonance) and EIMS (electron impact mass spectrometry) data with those from the literature [31]. This compound spontaneously forms a translucent crystal analyzed by X-ray diffraction, which confirmed the interpretation of NMR data.

Chloroform extracts of the fruit endocarp exhibited on TLC the presence of nine layers under UV light (254 nm), with Rf values of 0.187, 0.242, 0.286, 0.341, 0.396, 0.462, 0.659, 0.769, and 0.846, respectively, when a mobile phase of chloroform: ethyl acetate: diethyl ether (40:40:20) was used. After spraying with vanillin sulfuric acid, the stains observed here were pink and dark pink for most of them, which indicates the presence of phenols or steroids. The main compound (3 mg) with a Rf value of 0.187 was collected, redissolved, filtered, and isolated to yield dihydroxyacidissimol (C_25_H_34_NO_5_, molecular mass 427.2359 g/mol) by comparing its ^1^H-NMR and EIMS data with those from the literature [41,42].

### 2.8. In Silico Studies—Auto Dock Vina (Blind Docking Methodology)

#### 2.8.1. Protein Preparation

##### Main Protease

We took Mpro (Pdb: 6LU7 with 2.16 Å resolution), which has 306 amino acid residues, as our target protein, which contained a bound inhibitor known as N3 in its crystal structure [43]. For docking preparation, we removed the water molecules from the crystallographic structure of Mpro and removed the N3 molecule as well. Thus, ligands can be docked within every pocket of the protein. Next, we added a polar hydrogen atom because crystallographic structures usually lack hydrogen atoms. The addition of polar hydrogen atoms and removals of the water molecules and N3 were done with Pymol software [44]. Then, the protein molecule was saved in pdb format.

##### Spike Protein Receptor-Binding Domain (S-RBD) Bound with the ACE2 Complex

The SARS-CoV-2 S-RBD bound with the ACE2 complex X-ray diffraction structure (PDB: 6LZG with 2.50 Å resolution) was downloaded from the protein data bank (6LRG. Available online: https://www.rcsb.org/structure/6LZG (accessed on 1 January 2022)). This complex structure has two protein chains (Chain A is ACE2 and Chain B is S-RBD)

##### Spike Protein Receptor-Binding Domain (S-RBD)

The S-RBD structure was extracted from a SARS-CoV-2 S-RBD bound with the ACE2 complex X-ray diffraction structure (PDB: 6M0J with 2.45 Å resolution) (6M0J. Available online: https://www.rcsb.org/structure/6M0J (accessed on 1 January 2022)). Here, we took only the S-RBD as our target receptor and removed the ACE2 protein chain.

##### Cathepsin L

The X-ray crystal structure of cathepsin L was found in the PDB (PDB Id: 3HHA with 1.27 Å resolution) [45]. The crystal structure has four identical protein chains. We took the monomeric form of cathepsin L.

##### Nsp13 Helicase

Crystal structure of SARS-CoV-2 helicase at 1.94 Å (PDB:6ZSL) was downloaded from pdb (6ZSL. Available online: https://www.rcsb.org/structure/6ZSL (accessed on 1 January 2022)) [46].

#### 2.8.2. Ligand Preparation

Ligand molecules were downloaded from PubChem [47] in sdf format. They were optimized with the force field type MMFF94 using Openbable software and saved in pdbqt format.

##### Docking

We have used here the blind docking method for screening phytochemicals. So, the grid box in Autodock Vina was generated, aiming to cover up the whole protein molecule. In which region the ligand binds effectively with protein molecule can be found in blind docking. We have used exhaustively “16” for better ligand and protein binding. AutoDock Vina tool [48] provides a total of nine docked poses for each ligand; among them, pose1 is the best pose with the highest binding affinity. We have saved pose1 in pdb format by using Pymol for further analysis. 2D diagrams and the interactions between the ligand and amino acids of the protein were obtained in Discovery Studio Software [49].

### 2.9. Statistical Analysis

All values presented in the results section are the mean or mean ± standard deviation of the mean of three independent analyses, calculated using GraphPad Prism v6 Software (GraphPad Software Inc., La Jolla, CA, USA). Interpolated sigmoidal curves (4-parameter logistic curve, 4 PL) were determined automatically using GraphPad Prism v6 software (GraphPad Software Inc., La Jolla, CA, USA).

## 3. Results

### 3.1. Plant Extraction

The air-dried parts of the plant were extracted successively with hexane, chloroform, and methanol, respectively, to obtain lipophilic (non-polar), amphiphilic (mid-polar), and hydrophilic (polar) extracts from *B. malaccensis*. The average yield values ranged from 1.0 to 9.5% (Table 1). The average yields calculated for the hexane, chloroform, and methanol extracts were 2.7, 4.9, and 2.3%, respectively.

Chloroform fruit endocarps extract gave the highest extraction yield (9.5%), while the same plant part extracted with methanol yielded the lowest (1%).

### 3.2. Broth Microdilution

The broth microdilution method [38] was used to determine the minimum inhibitory concentration (MIC) of extracts against a panel of five bacteria (Table 2). The broth microdilution assay results confirmed that Gram-positive bacteria were more susceptible than Gram-negative bacteria to *B*. *malaccensis* extracts. The chloroform extract of leaves exhibited the lowest MIC against *S*. *aureus* and *B. subtilis*, with values of 250 µg/mL and a minimum bactericidal concentration (MBC) above 1000 µg/mL. The lowest MICs against *E*. *coli* and *P*. *aeruginosa* were observed with the methanol extract of bark (500 and 250 µg/mL, respectively) and MBC value above 1000 µg/mL. This extract inhibited the growth of *S*. *aureus* with MIC/MBC values of 250/1000 µg/mL, and, as such, it had the broadest spectrum of activity out of the 18 extracts tested. None of the extracts was active against *A*. *baumannii* (imipenem-resistant).

### 3.3. Antibiotic-Potentiating Activities

Of the 18 extracts tested, the most effective antibiotic-potentiator for Gram-positive bacteria was the methanol extract of wood with the β-lactam amoxicillin against *S*. *aureus* (Table 3). Regarding Gram-negative bacteria, the hexane and chloroform extracts of wood potentiated the aminoglycoside gentamicin against *E*. *coli*, with increments of inhibition zones of about 8 and 10 mm, respectively. We observed that the methanol extract of endocarps was able to rend penicillin G active against *E*. *coli* and the chloroform extract of endocarps relinquished the resistance of clinical isolates of *A*. *baumannii* to imipenem. In addition, the hexane extract of seeds acted potentiator for the quinolones (ciprofloxacin and levofloxacin) action against *P*. *aeruginosa*.

### 3.4. Cytotoxic Activities

**Figure 1:**
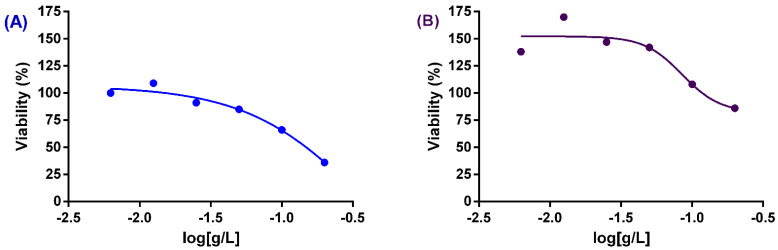
Cytotoxicity of chloroform (**A**) and methanolic (**B**) extracts of pericarps of *B*. *malaccensis* using Sigmoidal 4PL dose-response curves.

The toxicity of the 18 extracts against MRC-5 (Medical Research Council cell strain 5) human fibroblast cells was evaluated. The lowest 50% cytotoxic concentration (CC_50_) was obtained with the chloroform extract of pericarps, with a value of 0.36 g/L (Figure 1A). The methanolic extract pericarps exhibited a CC_50_ value of 0.09 g/L (Figure 1B).

### 3.5. Isolation of the Main Constituents from Active Extracts and Antibacterial Effects

**Figure 2:**
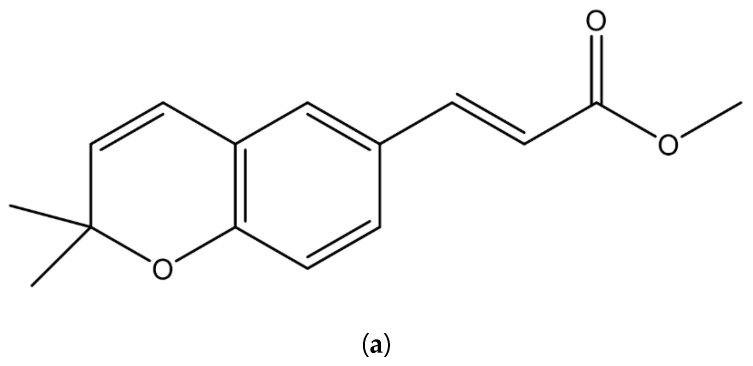

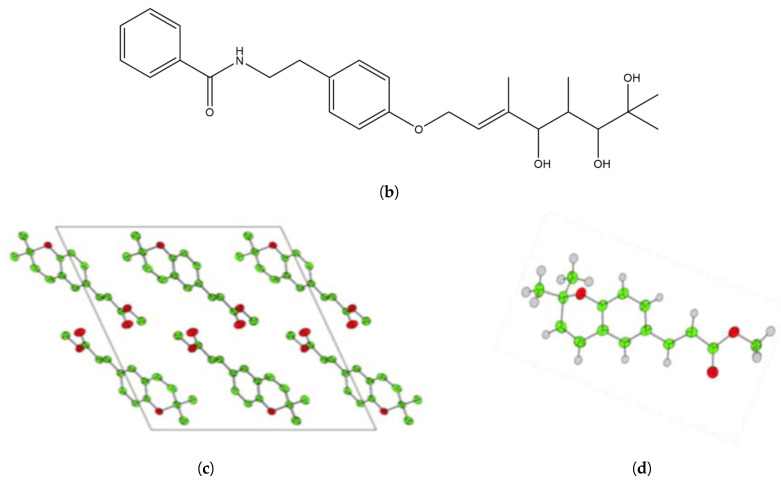
(**a**) Werneria chromene; (**b**) Dihydroxyacidissiminol; (**c**,**d**) Crystal structure of werneria chromene; (**c**) Cell packing in monoclinic state; (**d**) Crystal structure of isolated methyl (Z)-3-(2,2-dimethyl-2H-chromen-6-yl) acrylate from the ORTEP diagram at 50% ellipsoid probability.

The hexane extract of seeds that increased the potencies of quinolone antibiotics against *P. aeruginosa* was subjected to preparative TLC, yielding werneria chromene from the extract (Figure 2a, Table 4). From the chloroform extract of endocarps, the tyramine alkaloid dihydroxyacidissiminol was isolated (Figure 2b; Table 5). The absolute structure of werneria chromene was further confirmed using X-ray diffraction (Figure 2c,d). Werneria chromene was inactive against all bacteria tested except *P*. *aeruginosa*, with the MIC value of 1000 µg/mL and the MBC value of 1000 µg/mL, whereby both compounds repressed the growth of *P*. *putida* the MIC value of 1000 µg/mL and the MBC of 1000 µg/mL.

### 3.6. Crystal Structure of Isolated Methyl (Z)-3-(2,2-dimethyl-2H-chromen-6-yl) Acrylate Werneria Chromene

During the isolation process of werneria chromene, translucent crystals were obtained, and its chemical structure was confirmed by X-ray diffraction as methyl (E)-3-(2,2-dimethyl-2H-chromen-6-yl) acrylate. The crystal structure (Figure 2c,d) of werneria chromene indicated that the core structure is based on 2H-chromene, known as benzopyran, whereas the cyclic pyran ring takes the half boat conformation shape. The bond length of C8-O3 at 1.367 Ǻ is shorter than C14-O3 at 1.465 Ǻ, which forms the fused pyran. A planar geometry is exerted throughout the chemical structure while being extended by the side group in the form of methyl ester. Configuration E allows the structure to take a highly conjugated property, as the 10-atom chain packing of werneria chromene is monoclinic in space group P 1 21/c 1, as shown in Figure 2c,d. The core structure of benzopyran exists in cyclin-dependent kinase (CDK) inhibitors and can be found in drugs such as flavopiridol (also known as alvocidib) [50]. Werneria chromene does not have a chiral center and thus does not have conformers.

### 3.7. In Silico Studies with Werneria Chromene and Dihydroxyacidimissinol

**Figure 3:**
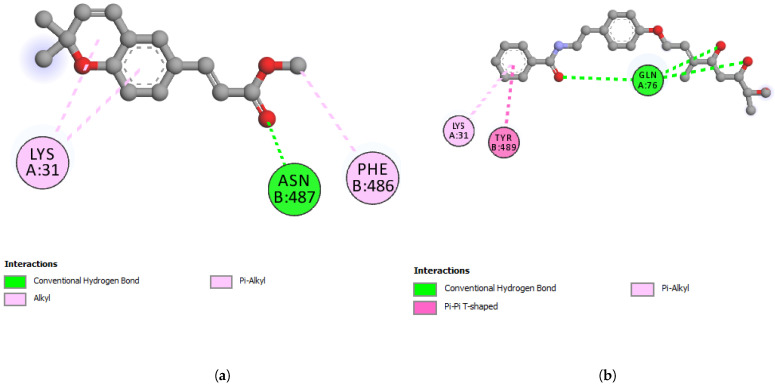
2D-interactions between (**a**) werneria chromene and S-RBD-hACE2; (**b**) dihydroxy acidissiminol and S-RBD-hACE2.

The reported structure of the SARS-CoV-2 spike protein receptor-binding domain complex with human ACE2 (S-RBD-hACE2) (PDB ID: 6LZG) was used for docking studies with werneria chromene and dihydroxyacidimissinol (2D interactions shown in Figure 3a,b, respectively). For cathepsin L, the reported structure PDB ID: 3HHA was used. PDB ID: 6ZSL was used for NSP13 helicase; PDB ID: 6LU7 was used for Mpro; and PDB ID: 6M0J was used for the spike protein receptor-binding domain (S-RBD). The binding energies of the two compounds (ΔG = kcal/mol) to the target proteins are shown in Table 6.

**Figure 4:**
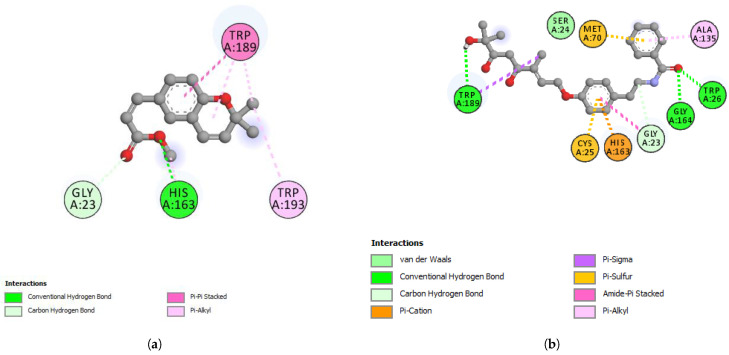
(**a**) 2D-interaction between werneria chromene and cathepsin L and S-RBD-hACE2; (**b**) 2D-interaction between dihydroxyacidissiminol and cathepsin L.

Werneria chromene did not display a good binding affinity to any of the five target proteins. The least predicted binding energy was observed with 6LZG (S-RBD-hACE2) and showed the predicted binding energy (ΔG) of −6.6 kcal/mol. On the other hand, dihydroxyacidissiminol showed the predicted binding energies of −8.1, −7.6, −7.0, and −7.5 kcal/mol with cathepsin L, nsp13 helicase, Mpro, and S-RBD, respectively (Table 6 and Table 7). Cathepsin L (PDB ID: 3HHA) with 220 amino acid residues has a two-chain form, R and L. The L domain contains 3 α-helices, while the R domain is a β-barrel closed at the bottom by a α-helix. The reactive site comprises His163 located at the top of the β-barrel and Cys25, which is located at the N-terminus of the central helix in the L domain [51]. The interacting amino acid residues of cathepsin L, forming hydrogen, hydrophobic, or electrostatic bonds with dihydroxyacidissiminol, include Gly23, Cys25, Ser24, Trp26, Met70, Ala135, His163, Gly164, and Trp189. The involvement of dihydroxyacidissiminol in interacting with both reactive site amino acids Cys25 and His163 makes this compound a potential potent inhibitor for cathepsin L. The 2D interactions of cathepsin L with werneria chromene and dihydroxyacidissiminol are shown in Figure 4a,b, respectively. PDB ID: 6ZSL represents the Nsp13 helicase of SARS-CoV-2. The structure of Nsp13 of SARS-CoV-2, like that of SARS-CoV, shows five domains, namely, RecA-like domains 1A and 2A, the 2B domain, the zinc-binding domain (ZBD), and the stalk domain. 

**Figure 5:**
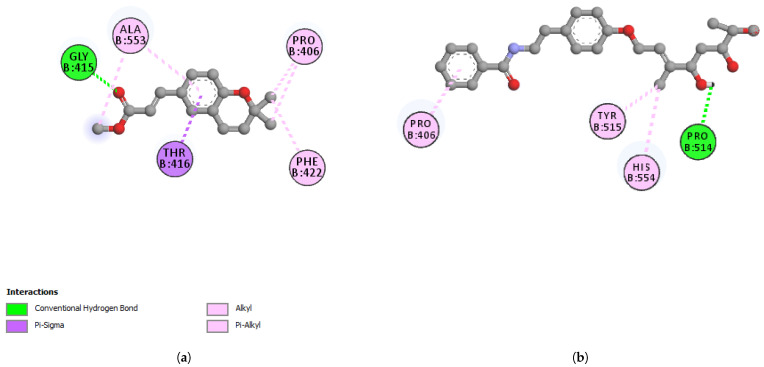
(**a**) 2D-interaction between werneria chromene and Nsp13 helicase; (**b**) 2D-interaction between dihydroxyacidissiminol and Nsp13 helicase.

The key amino acid residues of the ATP-binding site are six in number and are Lys288, Ser289, Asp374, Glu375, Gln404, and Arg567 [52]. Dihydroxyacidissiminol showed the predicted binding energy of −7.6 kcal/mol with Nsp13 helicase. The interacting amino acids of Nsp13 helicase, forming hydrophobic and hydrogen bonds with dihydroxyacidissiminol, were found to be Pro406, Pro514, Tyr515, and His554. Interestingly, none of the interacting amino acid residues of Nsp13 helicase with dihydroxyacidissiminol were from the ATP-binding site. Further studies are therefore needed to determine whether the binding of dihydroxyacidissiminol to Nsp13 helicase will lead to inhibition of helicase activities or not. What is noteworthy is that dihydroxyacidissiminol interacts with the C-terminus domain of the Nsp13 helicase, which is necessary for its helicase activities. The 2D interactions of Nsp13 helicase with werneria chromene and dihydroxyacidissiminol are shown in Figure 5a,b, respectively. The spike protein (S) of SARS-CoV-2 comprises two subunits S1 and S2, and the first subunit is responsible for binding to its receptor hACE2. Human ACE2 has two hotspots for the receptor-binding domain (RBD) of S, hotspot 31 and hotspot 353. SARS-CoV-2 recognizes hACE2 hotspot 31 through two amino acid residues on its RBD, Gln493 and Leu 455.

**Figure 6:**
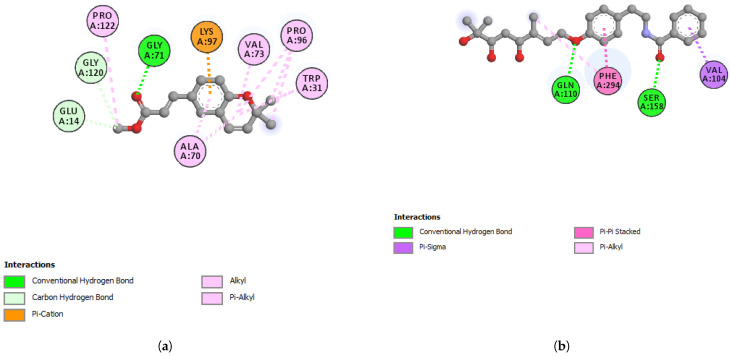
(**a**) 2D-interaction between werneria chromene and Mpro; (**b**) 2D-interaction between dihydroxy acidissiminol and Mpro.

**Figure 7:**
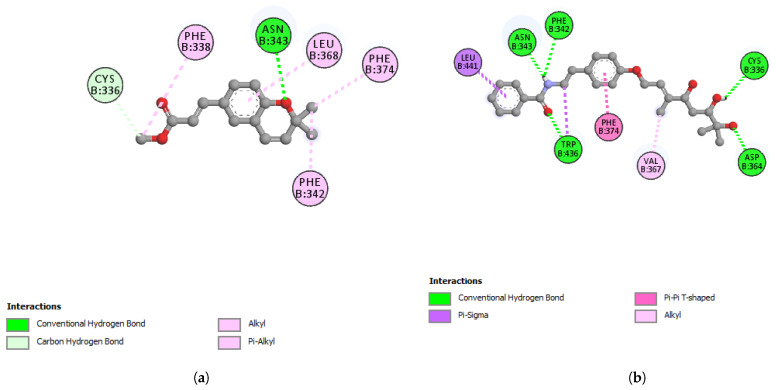
(**a**) 2D-interaction between werneria chromene and S-RBD; (**b**) 2D-interaction between dihydroxyacidissiminol and S-RBD.

Other amino acid residues playing significant roles in RBD interactions with hotspots 31 and 353 include Phe486, and Ser494 [53]. Dihydroxyacidissiminol interacts through hydrogen and hydrophobic bonding with amino acid residues Cys336, Phe342, Asn343, Asp364, Val367, Phe374, Trp436, and Leu441 of S-RBD. The S-RBD of SARS-CoV-2 comprises amino acid residues 387-516 [54]. Apart from the last two amino acids of S-RBD, dihydroxyacidissiminol shows interactions with other amino acid residues outside S-RBD. The conclusion formed is that despite showing a high binding affinity for S-RBD, dihydroxyacidissiminol possibly will have little or no inhibitory influences on S-RBD binding to hACE2. Both phytochemicals werneria chromene and dihyroxyacidissiminol did not show predicted good binding energies to Mpro. The 2D interactions of werneria chromene and dihydroxyacidissiminol with Mpro are shown in Figure 6a,b, respectively; the 2D interactions of werneria chromene and dihydroxyacidissiminol with S-RBD are shown, respectively, in Figure 7a,b. Taken cumulatively, dihydroxyacidissiminol shows predicted low-binding energies for cathepsin L. Since it interacts with both reactive site amino acids Cys25 and His163, it makes this compound a potential potent inhibitor for cathepsin L. The protease (cathepsin L or CTSL) plays a major role in SARS-CoV-2 infectivity. The circulating level of CTSL increases after SARS-CoV-2 infection and is positively correlated with disease course and severity. Scientists have postulated that the enzyme can make a good therapeutic target [36].

## 4. Discussion

COVID-19 caused by SARS-CoV-2 is the first but possibly not the last zoonotic virus that can paralyze global human activities. This pandemic reminded researchers to be vigilantly prepared to protect human lives from any potential viral or bacterial infections that may lead to cause a pandemic-like situation. Various phytochemicals from rainforests can be investigated in search for potential drugs to prevent these infections, although these are yet to validate the speculation. However, human activities, such as the increased need of food for the global growing population and industrialization, lead to deforestation. The Malaysian primary rainforest may disappear in the face of intense logging and palm-oil plantations, which has claimed around 1.1 million hectares of rainforest between 1990 and 2005 [55]. It must be grasped that the disappearance of primary rainforest trees signifies the disappearance of potential drugs [56]. *B*. *malaccensis* was collected in the primary rainforest (one of the few remaining pockets of the primary rainforest) of Manong, located in the north of Peninsular Malaysia, on the banks of the Perak River. This plant belongs to the subfamily Aurantioideae and the tribe Citrinae [57]. We selected that plant because (i) it was having fruits and flowers, allowing botanical identificationl (ii) it belongs to the family Rutaceae, which is a rich source of antimicrobial compounds; and (iii) and it is traditionally used by the Malays of Perak as medicine. Members of the Rutaceae family have been reported to have antibacterial, antibiotic-potentiating, and anti-viral properties [29,30,31,32]. Since there has been no study thus far to investigate the antimicrobial activities of *B*. *malaccensis*, its antibacterial and synergistic antibacterial, as well as its potent anti-viral properties against SARS-CoV-2 were explored. Some plants from the Rutaceae family, including *Citrus* *sinensis* (L.) Osbeck, have been used for the treatment of flu in traditional medicine and have antimicrobial effects [33]. *Citrus* *limon* (L.) Osbeck, *C. sinensis*, and *Citrus* *pardisi* Macfad. from Rutaceae reportedly showed significant activity against Hepatitis A virus (HAV) [28]. It is important to note that naringenin, a flavanone almost exclusively found in members of the genus *Citrus* L. fruits, has been reported to be a potent inhibitor of SARS-CoV-2 [34]. The average yield values ranged from 1.0 to 9.5%, indicating a fair extraction process [58,59].

First, the antibacterial activities of 18 extracts were assessed by a microbroth dilution assay against a panel of Gram-positive and Gram-negative bacteria and most of these extracts displayed levels of inhibition, especially the chloroform extract of leaves and methanol extract of bark, as these had the highest potencies; this result is reported for the first time. The β-lactam antibiotic imipenem is one of the last-resort treatments for *A. baumannii* infections [60]. Outbreaks of nosocomial infection caused by *A. baumannii* resistant to imipenem have reached the proportions of a global health emergency [52]. According to the International Network for the Study and Emergency Prevention of Antimicrobial Resistance [61], multi-resistant *A. baumannii* infection is a “sentinel event that warrants a coordinated response to control this multi-resistant pathogen” [62]. Gram-positive bacteria were more sensitive to the extracts than Gram-negative bacteria, in line with the literature [63]. Gram-positive bacteria are also more susceptible to xenobiotics since they only have a peptidoglycan wall, which is not an effective permeability barrier compared to Gram-negative bacteria, and are equipped with an outer lipopolysaccharide layer, porins, and an arsenal of efflux pumps. *P*. *aeruginosa* resists most antibiotics via β-lactamases, efflux pumps, loss, or alteration of the outer membrane porin [64]. *P. aeruginosa* is also resistant to fluoroquinolone exposure through mutations in their DNA gyrase and topoisomerase IV enzymes, as well as in efflux pumps [65].

The MIC of *B*. *malaccensis* extracts was determined by the microdilution broth assay. Rios and Recio suggested that a crude extract with MIC greater than 1000 µg/mL is inactive and proposed interesting antibacterial activity for MICs of 100 µg/mL or lower [66]. Earlier, Fabry and colleagues have defined crude active extracts as having MIC values below 8000 µg/mL [67]. While, more recently, Kuete used stricter endpoint criteria, in which crude extracts with MIC values less than 100 µg/mL are considered active [68]. Further, Kuete classified MICs above 625 µg/mL as weakly active extracts [68]. Following Kuete (2010) reports, it can be said that chloroform extract from *B. malaccensis* leaves exhibited mild antibacterial effects against the two Gram-positive bacteria tested (*S. aureus* and *B. Subtilis*) [69]. Moreover, extracts of antibacterial compounds can be categorized into two classes: bacteriostatic (MBC/MIC ratio greater than 4) and bactericidal (MBC/MIC ratio less than or equal to 4), according to Krishnan et al. [27]. Following this classification, the chloroform extract of leaves was bacteriostatic, while the methanol extract of bark was mildly bacteriostatic against *S. aureus* and *P. aeruginosa*. None of the plant extracts were active against *A*. *baumannii* (imipenem-resistant).

Regarding the antibiotic-potentiating activity of the *B*. *malaccensis* extracts tested, the strongest potentiation for Gram-positive bacteria was observed with the methanol extract from wood (0 mm) with amoxicillin (16 ± 0.0 mm) against *S. aureus* (23.7 ± 0.5 mm). Of note, it was observed that the chloroform extract of endocarps was able to render penicillin G active against *E. coli* and potentiated imipenem activity against imipenem-resistant *A. baumannii*. The hexane extract of *B*. *malaccensis* seeds also potentiated the antibiotic activities of ciprofloxacin and levofloxacin against the multidrug-resistant bacteria *P. aeruginosa*. These antibiotic-potentiating properties are reported for the first time. The bacterial strains we tested are, except for imipenem-resistant *A. baumannii*, antibiotic sensitive, and the extract tested further increased their vulnerability to antibiotics.

In this study, the strongest potentiation was observed with the methanol extract of wood with the β-lactam antibiotic amoxicillin towards *S. aureus*. It is an important finding because amoxicillin resistance represents a severe clinical burden worldwide in hospitals [70]. *S. aureus* resists amoxicillin via β-lactamases and changes in penicillin-binding protein 2a [71]. The low standard deviation obtained indicate that data are close to the mean. The hexane extract of the seeds potentiated ciprofloxacin and levofloxacin against *P. aeruginosa*. The nosocomial bacterium *A. baumannii* resists imipenem via intrinsic and acquired metallo-β-lactamases and oxacillinases, as well as porin loss [43]. In China, for instance, more than 50% of isolates were found to be imipenem-resistant in 2009, and in Thailand, the rate of resistance to imipenem increased significantly from 2% in 2000 to 67% in 2011 [72]. *E. coli* is known to resist penicillin G via penicillin acylase and β-lactamases [73], as well as efflux pumps [74].

Cell line cytotoxicity (at concentrations of 0-200 µg/mL of extracts against human MRC-5 cells) was assessed following the confirmed antibacterial activities of some *B*. *malaccensis* extracts. The American National Cancer Institute defines a plant extract as toxic to human cells when the CC_50_ values are below 30 µg/mL after an exposure time of 72 h [75]. Accordingly, none of the extracts studied were toxic in vitro for the cell line tested in this study. Among all tested extracts, the chloroform extract of *B. malaccensis* demonstrated the lowest cytotoxicity. Additionally, the identification of the main constituents of the hexane extract of seeds and the chloroform extract of fruits was carried out, which elicited interesting antibiotic-potentiating effects against problematic Gram-negative bacteria, resulting in the isolation and characterization of werneria chromene and dihydroxyacidissiminol.

Since werneria chromene was selectively active against *P. aeruginosa*, its activity against *P. putida* and observed and activity was observed. Likewise, hydroxyacidissimol was specifically active against *P. putida*. These activities, although weak, are specific and are reported for the first time, although the reasons for this specificity against *Pseudomonas* spp. remain unknown. We did not attempt to isolate compounds from methanol extracts as we looked for mid-polar to non-polar compounds that may have better ADME.

The occurrence of these constituents in *B. malaccensis* was not known previously. Both compounds were weakly but specifically bacteriostatic against *P*. *putida* and inactive for all other bacteria tested. Further, the crystal structure of werneria chromene is reported here for the first time. However, their synergistic activities were not examined due to insufficient amounts of available extracts, which requires further investigation. We are currently examining minor constituents in these extracts and found a series of prenylated flavonols (unpublished data) that may work synergistically to bring about antibiotic-potentiating effects and we are looking into this matter.

In silico molecular docking of werneria chromene and dihydroxyacidissimol was examined with the reported structure of the SARS-CoV-2 spike protein receptor-binding domain complexed with human ACE2 (S-RBD-hACE2) (PDB ID: 6LZG), human cathepsin L (PDB ID: 3HHA); PDB ID: 6ZSL was used for NSP13 helicase; PDB ID: 6LU7 was used for Mpro; and PDB ID: 6M0J was used for the spike protein receptor-binding domain (S-RBD) [50,76,77,78,79]. The 6M0J is also a spike protein RBD bound with ACE2, but unlike 6LZG ACE2 was removed from the spike protein RBD and molecular docking studies conducted with the spike protein RBD only. Dihydroxyacidissiminol showed a good affinity for essential target proteins (i.e., its binding energy values were −8.1, −7.6, and −7.5 kcal/mol for cathepsin L, nsp13 helicase, and spike protein receptor-binding domain, respectively) associated with SARS-CoV-2 entry and replication in human cells, such as spike protein receptor-binding domain (S-RBD), cathepsin L, and Nsp13 helicase.

## 5. Conclusions

The decrease in the development of new and effective antibiotics by pharmaceutical industries and the concomitant and steady increase in bacterial resistance leaves clinicians with the increasing difficulty to save the life of patients infected by nosocomial bacteria globally. As the world is going through a pandemic caused by SARS-CoV-2, a part of the mortality is derived from SARS-CoV-2 infection associated with bacterial infections. The development of resistance-modifying agents can be an additional strategy to overcome bacteria multidrug resistance. To our knowledge, the antimicrobial effect potentiating properties of *B*. *malaccensis* have not been previously reported. Most of the extracts demonstrated inhibiting the growth of Gram-positive bacteria in particular. Two compounds, werneria chromene and dihydroxylacidissiminol, isolated from this plant’s extracts, inhibited the growth of *P. putida*, and dihydroxyacidissiminol demonstrated a good affinity for cathepsin L. The amount of available plant was a limitation to our study, and we plan to obtain larger collections. The principle involved in antibiotic-potentiation, the in vitro activity of dihydroxyacidissiminol against SARS-CoV-2 and other coronaviruses, and the therapeutic potential of the compound isolated as specific inhibitors of *Pseudomonas* spp. need to be examined.

## Figures and Tables

**Table 1:** Percentage extraction yields of 18 organic extracts from *B. malaccensis*.

Part Extracted	Plant Extracts Yield (%)		
	Hexane	Chloroform	Methanol
Leaves	1.8	3.3	1.9
Bark	3.8	5.4	1.2
Wood	1.0	3.4	4.2
Fruit pericarp	5.1	2.9	2.1
Fruit endocarp	3.4	9.5	1.0
Seeds	1.5	5.1	3.6
Average yields	2.7	4.9	2.3

**Table 2:** Minimum inhibitory concentration (MIC) by broth microdilution (µg/mL).

Plant Part	Solvent	*S. aureus* (ATCC 11632)	*B. subtilis* (ATCC 6633)	*E. coli* (ATCC 8379)	*P. aeruginosa* (ATCC 10145)	*A. baumannii* (Imipenem-Resistant)
Leaves	Hexane	1000	-	-	-	-
Leaves	Chloroform	**250** (>1000)	**250** (>1000)	-	-	-
Leaves	Methanol	500	1000	-	-	-
Bark	Hexane	-	-	-	1000	-
Bark	Chloroform	1000	-	-	-	-
Bark	Methanol	**250** (>1000)	-	500	**250** (>1000)	-
Wood	Hexane	625	1250	5000	2500	-
Wood	Chloroform	2500	2500	2500	625	-
Wood	Methanol	-	2500	2500	2500	-
Endocarp	Chloroform	-	-	-	1000	-
Endocarp	Methanol	-	-	-	1000	-
Seeds	Hexane	-	-	-	1000	-
Chloramphenicol		0.03	0.02	Nt	Nt	Nt
Tetracycline		Nt	Nt	0.02	0.01	-
Imipenem Negative control		Nt Fg	Nt Fg	Nt Fg	Nt Fg	12.0 Fg

Abbreviations: Nt, Not tested; Fg, Full bacterial growth; ‘-’, No activity. Extracts with no activity against all the bacteria tested are not included here in this table. Bold data indicate the lower MIC values. Values are given as the mean of triplicates. Second values in parentheses represent corresponding minimum bactericidal concentrations (MBC).

**Table 3:** Antibiotic-potentiating activities (mm).

Treatment with	*S. aureus* (ATCC 11632)	*B. subtilis* (ATCC 6633)	*E. coli* (ATCC 8379)	*P. aeruginosa* (ATCC 10145)	*A. baumannii* (Imipenem-Resistant)
Extracts			-	-	-
I	-	7.0 ± 1.4	-	-	-
II	12 ± 0.0	-	-	-	-
III	-	-	-	-	-
IV	-	-	-	-	-
V	-	-	-	-	-
VI	-	-	-	-	-
VII	-	-	-	-	-
Amoxicillin	16 ± 0.0	14.3 ± 0.5	-	-	-
Ampicillin	41 ± 0.3	20 ± 0.1	-	-	-
Ciprofloxacin	-	38 ± 0.0	-	35 ± 0.02	-
Gentamicin	-	-	25 ± 0.01	-	-
Levofloxacin	-	35 ± 1.0	38 ± 0.2	28 ± 0.3	-
Penicillin G	-	-	-	-	-
Imipenem	-	-	-	-	10 ± 0.04
Amoxicillin + I	-	26.7 ± 0.0	-	-	-
Amoxicillin + II	22.5 ± 0.5	-	-	-	-
Amoxicillin + III	**23** **.** **7 ± 0** **.** **5**	-	-	-	-
Ampicillin + II	40.7 ± 0.8	-	-	-	-
Ampicillin + III	**42** **.** **0 ± 0** **.** **5**	-	-	-	-
Ampicillin + IV	-	22 ± 0.3	-	-	-
Ciprofloxacin + IV	-	39 ± 0.1	-	**38 ± 1** **.** **1**	-
Ciprofloxacin + V	-	38.5 ± 0.0	-	36 ± 0.1	-
Gentamicin + I	-	-	**34** **.** **3 ± 1** **.** **7**	-	-
Gentamicin + II	-	-	**35** **.** **3 ± 1** **.** **3**	-	-
Levofloxacin + IV	-	-	-	**30.7 ± 0** **.** **6**	-
Levofloxacin + V	-	38.5 ± 0.3	-	30 ± 1.0	-
Penicillin G + VI	-	-	**6** **.** **5 ± 0** **.** **02**	-	-
Imipenem + V	-	-	-	-	**11 ± 1** **.** **2**

Abbreviations: I = Wood hexane (1 mg/disc); II = Wood chloroform (1 mg/disc); III = Wood methanol (1 mg/disc); IV = Seeds hexane; V = Endocarp chloroform; VI = Endocarp methanol; VII: Leaves chloroform. Amoxicillin (10 µg/disc); Ampicillin (10 µg/disc); Ciprofloxacin (5 µg/disc); Gentamicin (10 µg/disc0; Imipenem (10 µg/disc); Levofloxacin (5 µg/disc); ‘-’, No activity. Extracts without any synergy are not included. Synergies are indicated in bold. The values are expressed as the mean ± standard deviation.

**Table 4:** NMR data of werneria chromene (p.p.m.).

Position	*δ*-H [31]	δ-H Werneria Chromene	Integration	Position	δ-C [31]	δ-C Werneria Chromene
3	5.62 d	5,65 d	1	2	77.1	78
4	6.28 d	6.30 d	1	3	131.3	132
5	7.12 d	7.18 d	1	4	121.7	122
7	7.26 dd	7.25 dd	1	5	134.3	134
8	6.74 d	6.78 d	1	6	127.1	128
9	7.58 d	7.60 d	1	7	129.4	129
10	6.26 d	6.20 d	1	8	116.7	116
12,13	7.58 d	7.57 d	2,2	10	121.3	122
O-CH_3_	3.76 d	3.80 s	3	11	115	115
				12	144.6	145

**Table 5:** NMR data (p.p.m.) of dihydroxyacidissiminol.

Position	*δ*-H [42]	δ-H Dihydroxyacidissiminol	Integration	δ-C [42]	δ-C Dihydroxyacidissiminol
1′	-	-	-	134.60	135.00
2′,6′	7.69 d	7.70 d	1,1	126.80	126.50
3′,5′	7.41 t	7.40 t	1,1	128.60	128.90
4′	7.49 t	7.49 t	1	131.40	130.00
CO-NH	6.10 m	6.15 m	1	167.60	167.50
N-CH_2_	3.70 q	3.70 m	2	41.30	41.50
Ar-CH_2_	2.88 t	2.80 t	2	34.80	35.00
1″	-	-	-	157.30	157.00
2″, 6″	6.87 d	6.87 d	1,1	114.90	115.00
3″, 5″	7.18 d	7.16 d	1,1	129.80	129.90
4″	-	-	-	131.00	131.50
1	4.58 d	4.60 d	2	64.50	64.50
2	5.80 d	5.78 d	1	121.10	121.50
3	-	-	-	142.00	140.50
3-Me	1.76 s	1.75 s	3	12.40	12.90
4	4.35 dd	4.35 dd	1	77.40	79.00
5	-	-	-	-	-
6	3.64 m	3.65 m	1	78.70	77.50
7	-	-	-	72.60	74.00
7-Me	1.18 s	1.17 s	3	23.70	24.00
4-OH	1.55 s	1.75 s	-	-	-
6,7 OH	1.55 s	1.65 s	-	-	-

Abbreviation: ‘-’, No peak.

**Table 6:** Predicted binding energies (ΔG = kcal/mol) of werneria chromene and dihydroxyacidissiminol with various SARS-CoV-2 and human target proteins.

Phytochemical	Spike Protein RBD Bound with ACE2 PDB: 6LZG	Cathepsin L PDB: 3HHA	Nsp13 Helicase PDB: 6ZSL	Mpro PDB: 6LU7	Spike Protein RBD PDB: 6M0J *
Dihydroxyacidissiminol	−5.8	−8.1	−7.6	−7.0	−7.5
Werneria chromene	−6.6	−6.4	−6.4	−5.9	−6.0

* 6M0J is also spike protein RBD bound with ACE2, but unlike 6LZG, ACE2 was removed from spike protein RBD and molecular docking studies conducted with spike protein RBD only.

**Table 7:** Interaction of dihydroxyacidissiminol with amino acid residues of cathepsin L, Nsp13 helicase, and spike protein receptor-binding domain (S-RBD).

**Residue (Cathepsin L)**	**Distance**	**Category**	**Type**
**Dihydroxyacidissiminol**			
TRP26	2.68	Hydrogen Bond	Conventional Hydrogen Bond
GLY164	2.06	Hydrogen Bond	Conventional Hydrogen Bond
TRP189	2.10	Hydrogen Bond	Conventional Hydrogen Bond
GLY23	3.60	Hydrogen Bond	Carbon Hydrogen Bond
HIS163	3.64	Electrostatic	Pi-Cation
TRP189	3.67	Hydrophobic	Pi-Sigma
TRP189	3.99	Hydrophobic	Pi-Sigma
CYS25	4.81	Other	Pi-Sulfur
MET70	5.04	Other	Pi-Sulfur
GLY23, SER24	4.38	Hydrophobic	Amide-Pi Stacked
ALA135	4.15	Hydrophobic	Pi-Alkyl
**Residue (Nsp13 helicase)**	**Distance**	**Category**	**Type**
**Dihydroxyacidissiminol**			
PRO514	2.29	Hydrogen Bond	Conventional Hydrogen Bond
TYR515	4.64	Hydrophobic	Pi-Alkyl
HIS554	4.06	Hydrophobic	Pi-Alkyl
PRO406	4.13	Hydrophobic	Pi-Alkyl
**Residue (S-RBD)**	**Distance**	**Category**	**Type**
**Dihydroxyacidissiminol**			
ASP364	1.89	Hydrogen Bond	Conventional Hydrogen Bond
B:TRP436	2.29	Hydrogen Bond	Conventional Hydrogen Bond
CYS336	2.38	Hydrogen Bond	Conventional Hydrogen Bond
PHE342	2.70	Hydrogen Bond	Conventional Hydrogen Bond
ASN343	1.94	Hydrogen Bond	Conventional Hydrogen Bond
LEU441	3.58	Hydrophobic	Pi-Sigma
TRP436	3.98	Hydrophobic	Pi-Sigma
PHE374	4.75	Hydrophobic	Pi-Pi T-shaped
VAL367	4.58	Hydrophobic	Alkyl
